# Conservative Management of Dens Evaginatus: Report of Two Unusual Cases

**DOI:** 10.5005/jp-journals-10005-1067

**Published:** 2010-08-17

**Authors:** M Guna Shekhar, S Vijaykumar, J Tenny, GR Ravi

**Affiliations:** 1 Reader, Faculty of Pediatric Dentistry, Vishnu Dental College, Bhimavaram, Andhra Pradesh, India; 2 Reader, Conservative Dentistry and Endodontics, NIMS Dental College, Jaipur, Rajasthan, India; 3 Reader, Oral Medicine and Radiology, Vishnu Dental College, Bhimavaram, Andhra Pradesh, India; 4 Senior Lecturer, Faculty of Pediatric Dentistry, Vishnu Dental College, Bhimavaram, Andhra Pradesh, India

**Keywords:** Dens evaginatus, supernumerary tooth, lateral incisor, conservative therapy.

## Abstract

Dens evaginatus (DE) is a rare developmental anomaly characterized by presence of an extra cusp arising from occlusal or lingual surfaces. Isolated occurrence or concomitant presence of DE with other dental anomalies has been reported. DE commonly affects permanent teeth and is rarely seen in primary dentition. Treatment may be conservative or radical. This article presents two unusual cases of concomitant occurrence of a supernumerary tooth and DE affecting maxillary deciduous lateral incisor and conservative management of DE occurring concurrently with a possible Oehler’s type I invagination in maxillary permanent lateral incisor.

## INTRODUCTION

Disturbances during morpho-differentiation stage of tooth development have been related to abnormalities in the shape and size of a tooth. Dens evaginatus and dens invaginatus are developmental variations of the shape of a tooth.^1^ Dens evaginatus (DE) is a rare developmental defect involving exfolding of the enamel and dentin that takes the form of a tubercle, projecting from the occlusal or lingual surfaces of the affected tooth.^2^ The reported incidence varies from 1 to 4%, and the mongoloid and the Neo Asiaties racial groups are commonly affected, but have also been observed in Chinese and Caucasian people.^3^ This anomaly most frequently involves the premolars, but may also be seen in incisors, canines and molars, and occurs in both primary and permanent dentition.^4^

DE occurs normally alone, but can present itself in association with other dental anomalies such as supernumerary teeth, peg shaped incisors, bifid cingulum, and dens invaginatus.^5-7^ Other terms used to describe this anomaly include accessory cusp, leong’s premolar, evaginatus odontomas, occlusal pearl, and talon cusp (specifically for anterior teeth).^3,8^

While talon cusp originated as a descriptive term for DE due to its resemblance to an eagle’s talon, current concepts prefer the terminology DE, to describe this developmental aberration, as most authors agree that both DE and talon cusp share the same etiology occurring during the morpho-differentiation stage of tooth development.^8^ The evaginated tubercle usually is composed of normal enamel and dentin with varying extensions of pulp tissue, and is more prone to wear or fracture leading to early pulp pathosis. Treatment approaches to DE depends on the shape and size of the evagination and can be either conservative or radical.^9^

This article presents two unusual cases of concomitant occurrence of a supernumerary tooth and DE affecting maxillary deciduous lateral incisor and conservative management of DE occurring concurrently with a possible Oehler’s type I invagination in maxillary permanent lateral incisor.

## CASE REPORT

### Case 1

**Fig. 1:**
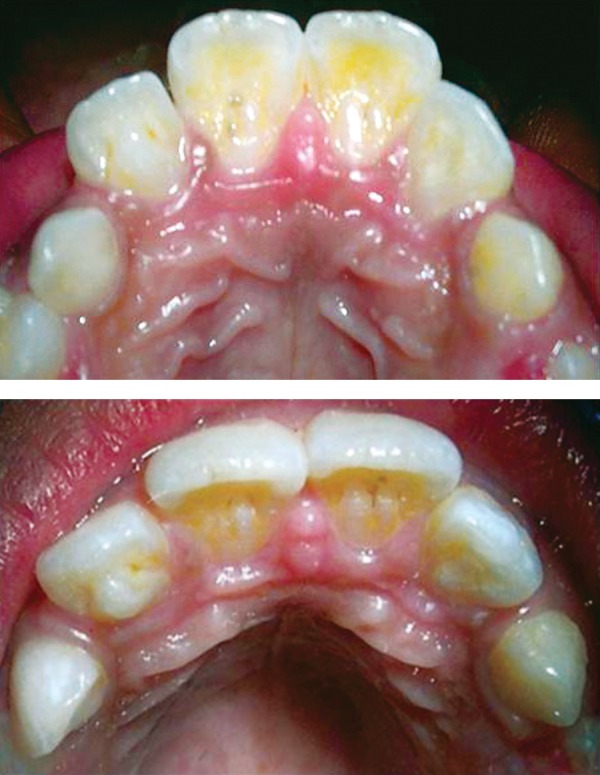
Occlusal and palatal views of left lateral incisor with extra cusp and central incisors with bifid cingulum in upper arch (mirror view)

A 9-year-old female came to the pediatric dental clinic for dental treatment. The medical and family history was noncontributory. Clinical examination disclosed carious lesions in primary and permanent molars, and a prominent cusp-like structure on the palatal surface of the maxillary left lateral incisor. It was pyramidal in shape and extended from CEJ half-way to the incisal edge causing minimal occlusal interference (Fig 1).

Clinically, the tooth was asymptomatic and responded normally to pulp vitality tests. The grooves at the junction of the cusp and palatal surface were stained and contained dental plaque. Maxillary right lateral incisor displayed a deep lingual pit suggesting type I invagination. Further, prominent bifid cingulae with deep stained fissures were apparent on the palatal aspects of both maxillary right and left central incisors. White spot lesions were visible in the invagination and around the tubercle (Fig. 1).

**Fig. 2:**
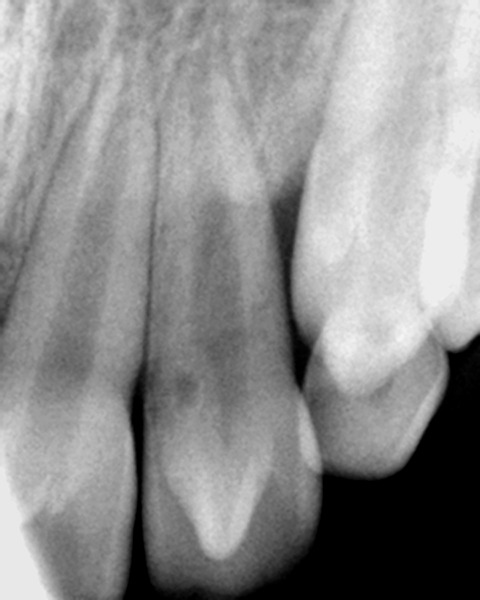
Periapical radiograph showing dens evaginatus on 22

A periapical radiograph showed a ‘V’ shaped radiopaque structure superimposed on the affected crowns, with the ‘V’ pointing towards the incisal edge. In addition, periapi-cal radiograph revealed an invagination lined by enamel, adjacent to the tubercle indicating probable Oehler’s type I invagination in 22. Radiographically no evidence of associated pathosis was found (Fig. 2).

The treatment plan consisted of oral hygiene instructions, restoration of carious teeth, and prophylactic restorative procedures in the maxillary central incisors and lateral incisors. The prophylactic treatment consisted of selective grinding of extra cusp with flare-shaped diamond bur under water coolant using high-speed hand piece at 3-month intervals.

**Fig. 3:**
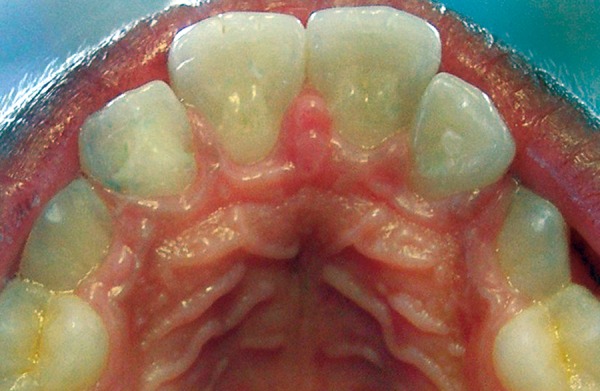
Post-treatment view (mirror view)

Each grinding session was followed by the application of a desensitizing/remineralizing agent with 0.2% fluoride (GC Tooth mousse plus, Recaldent, GC Co. Japan), to the surfaces of reduced cusp, after polishing, to reduce dentin sensitivity. The treatment lasted 12 months, during which preventive measures to caries were followed regularly.

Invasive sealing of the cusp-tooth intersection in incisors was planned. After prophylaxis of affected teeth, ameloplasty was performed on the grooves/fissures near the bifid cingulum using carbide bur in a high-speed hand piece and were then sealed with an acid-etch flowable composite resin to avoid penetration of irritants and microorganisms into the invagination (Fig. 3). After 18-month follow-up period, the tooth was still asymptomatic and responded normally to vitality tests. Patient is under regular clinical and radiographical re-evaluation to assess the occlusion and the progress of pulp recession.

### Case 2

**Fig. 4:**
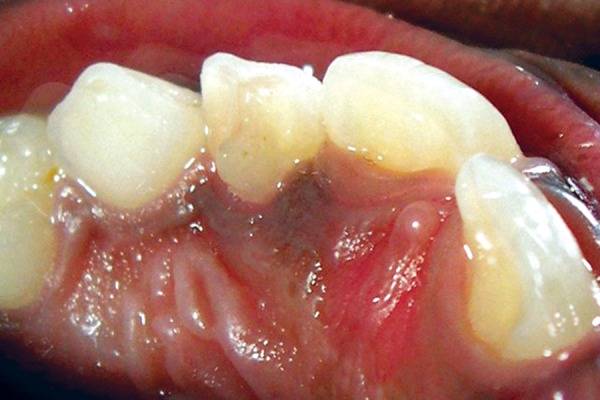
Palatal view showing eagle’s talon on 62 (mirror view)

A 6-year-old female presented for a routine dental examination. Her medical history was noncontributory and extraoral examination did not reveal any abnormalities. On intraoral examination, the left maxillary deciduous lateral incisor (62) was slightly mobile and revealed an extra cusp which extended from CEJ halfway to incisal edge, at right angles to the mesiodistal surface of the tooth crown on the lingual surface resembling an eagle’s talon, suggesting dens evaginatus. The cusp measured 2 to 2.5 mm in length (inciso-cervically), 1 to 1.5 mm in width (mesiodistally) and about 2 to 2.5 mm in thickness (labiolingually) approximately (Fig. 4).

**Fig. 5:**
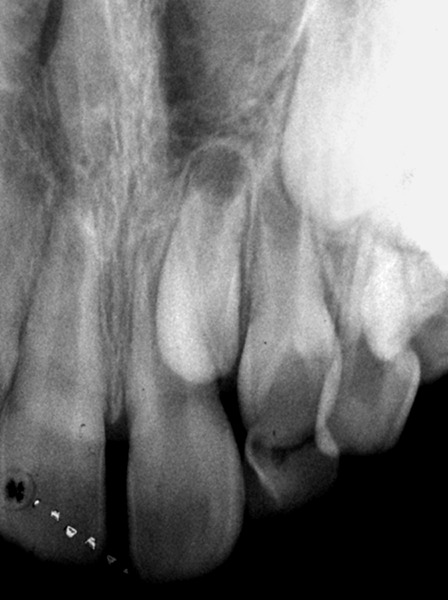
Periapical radiograph showing DE on 62 and an unerupted supernumerary tooth

She was born out of consanguineous marriage and family history was free of similar dental abnormalities. A periapical radiograph of 62 revealed a radiopaque structure superimposed on the crown. Further, the unerupted left maxillary permanent lateral incisor presented an invagina-tion like appearance lined by enamel with possible aspect of Oehler’s type I dens invaginatus. Additionally, an unerupted supernumerary lateral incisor (supplemental type) was noticed mesial to 62. No evidence of any periapical pathosis was evident radiographically (Fig. 5).

There were neither esthetic concerns nor clinical signs of any problems associated with deciduous maxillary lateral incisor. So no immediate dental intervention was advised and the patient is under regular follow-up.

## DISCUSSION

The exact etiology of these variations remains unclear. However, the probable role of genetics and environmental factors, such as trauma or other localized insults affecting the tooth germ have been suggested.^10^ In the two cases reported here, the etiology of dental abnormalities could not be clearly associated with any genetic or environmental factors, as the family history was negative in both the cases.

The case-2 presented here is unique because of the association of a supernumerary tooth and a probable dens invaginatus in 22, with DE in maxillary deciduous left lateral incisor. Only few cases of this anomaly in primary teeth have been reported in the literature.^5,6,11^ One plausible explanation for the association of extra cusp with a supernumerary tooth may be the local hyperactivity of dental lamina resulting in the occurrence of supernumerary and accessory cusps, during the bud-stage and morpho-differentiation stages, respectively.^5^

Small talon cusps are asymptomatic and require no intervention. Large talon cusps may cause clinical problems including occlusal interference, irritation of the tongue during speech and mastication, carious lesions in the developmental grooves that delineate the cusp, pulp necrosis, periapical pathosis, attrition of the opposing tooth, and periodontal problems due to excessive occlusal forces.^12^

On the contrary, the invagination in DI frequently communicates with the oral cavity, allowing the penetration of irritants and microorganisms into the pulp and may eventually lead to pulp necrosis. The treatment options for dens in-vaginatus include preventive sealing of the invagination, root canal treatment, endodontic apical surgery and extraction.^13^

The treatment of DE merits clinical consideration and objectives could include pulp vitality preservation, caries prevention, elimination of tongue irritation, and meeting esthetic and occlusal requirements.^4,14^ Treatment strategies are different for the deciduous and the permanent dentition. Oehlers and Colleagues (1967)^15^ advocated intermittent grinding of the tubercle to encourage reparative dentin formation. Myers ^16^ reported a case of talon cusp where in two-thirds of the cusp reduction was done followed by topical fluoride application at 4 month intervals over 5 years. Richardson (1985) ^17^ had suggested a preventive approach, which included pumice prophylaxis and acid-etching with phosphoric acid, followed by placement of a composite material.

Prophylactic treatment can be considered as the preferred mode of approach. Application of desensitizing/rematerial-izing agent containing 0.2% sodium fluoride following the gradual and periodic reduction of bulk of extra cusp, reduces sensitivity, stimulates reparative dentin formation, and allows the tooth to remain vital, especially in permanent teeth with open apices.^9^ Further, it increases tooth resistance to acid dissolution, promotes remineralization, and also inhibits the cariogenic microbial process.

In case-1 reported here, we have reduced 1 to 1.5 mm of extra cusp gradually on several consecutive visits at 3 month intervals, followed by application of a desensitizer (GC tooth mousse plus) to protect the pulp and allow deposition of reparative dentin. Here, the grinding procedure was carried out over 12 months. However, in case-2, no intervention was advised as the need for treatment in a primary talon-cusped tooth which will eventually exfoliate, other than for esthetic reasons, is low. Pit and fissure sealants are recommended as preventive measures, whilst composite resin and amalgam restorations have also been advocated as DE is prone to caries. Root canal therapy and extraction have been suggested in selected cases.^9^

## CONCLUSION

Dens evaginatus deserves clinical importance as it provides chances of early pulp pathosis. Radiologic examination plays a vital role in diagnosis in conjunction with clinical examination. Early and the correct diagnosis of dental abnormalities by clinicians are essential to preserve the vitality of the pulp.
